# DHA-supplemented diet increases the survival of rats following asphyxia-induced cardiac arrest and cardiopulmonary bypass resuscitation

**DOI:** 10.1038/srep36545

**Published:** 2016-11-04

**Authors:** Junhwan Kim, Tai Yin, Koichiro Shinozaki, Joshua W. Lampe, Lance B. Becker

**Affiliations:** 1 Laboratory for Critical Care Physiology, Department of Emergency Medicine, Feinstein Institute for Medical Research, Northwell Health System, Manhasset, New York, 11030, United States of America

## Abstract

Accumulating evidence illustrates the beneficial effects of dietary docosahexaenoic acid (DHA) on cardiovascular diseases. However, its effects on cardiac arrest (CA) remain controversial in epidemiological studies and have not been reported in controlled animal studies. Here, we examined whether dietary DHA can improve survival, the most important endpoint in CA. Male Sprague-Dawley rats were randomized into two groups and received either a control diet or a DHA-supplemented diet for 7–8 weeks. Rats were then subjected to 20 min asphyxia-induced cardiac arrest followed by 30 min cardiopulmonary bypass resuscitation. Rat survival was monitored for additional 3.5 h following resuscitation. In the control group, 1 of 9 rats survived for 4 h, whereas 6 of 9 rats survived in the DHA-treated group. Surviving rats in the DHA-treated group displayed moderately improved hemodynamics compared to rats in the control group 1 h after the start of resuscitation. Rats in the control group showed no sign of brain function whereas rats in the DHA-treated group had recurrent seizures and spontaneous respiration, suggesting dietary DHA also protects the brain. Overall, our study shows that dietary DHA significantly improves rat survival following 20 min of severe CA.

Cardiac arrest (CA) is a leading cause of death in the US, where the primary cause of mortality is ischemic damage to the brain and heart^1^,^2^,^3^,^4^. To improve survival rates, various controlled resuscitation methods have been tested in animal models and in clinical applications^5^,^6^. Although controlled resuscitation and the use of automated external defibrillators have shown improved survival and neurological outcomes in patients with CA^7^,^8^, the overall survival rate following CA remains lower than 10%^9^. Therefore, an increased focus on reducing damage during CA and/or reperfusion in addition to developing better resuscitation strategies is required.

Docosahexaenoic acid (DHA), an omega-3 fatty acid, is an essential nutrient for human health. Numerous clinical studies have examined the effects of dietary DHA on cardiovascular diseases including CA^10^. Despite growing evidence regarding the beneficiary effects of DHA on cardiovascular diseases, its effects on CA patient survival remain controversial. Early studies showed that intake of omega-3 fatty acids reduced the risk of CA^11^,^12^ as well as death rates from sudden CA^13^. However, Tavazzi *et al*. and Rauch *et al*. reported that an intake of n-3 fatty acids had no effect on the rate of sudden CA^14^,^15^. Moreover, Burr observed opposing results, where the consumption of fish or fish oil led to an increased rate of sudden cardiac death^16^,^17^.

Controlled animal studies have provided evidence that dietary DHA protects organs from ischemic damage. Studies using rodents have shown that dietary DHA reduces infarct size and enhances cardiac output in perfused ischemic hearts^18^,^19^ and decreases ischemic lesion size, edema, eosinophilic degeneration, and cellular inflammatory responses following brain ischemia^20^,^21^,^22^. Dietary DHA also reduces an inflammatory response in the liver^23^. However, whether the prevention of these biochemical and cellular responses in isolated ischemic organs can improve the function of organs in whole-body ischemia is still questionable. Furthermore, whether this protection can be further extended to the survival of animals after CA has not been examined.

In the current study, we tested whether dietary DHA is protective against CA using a rat model of asphyxia-induced CA followed by cardiopulmonary bypass (CPB) resuscitation. Asphyxia is the most reliable method to stop the heart from beating without generating non-ischemic injuries in rats^24^,^25^,^26^. CPB resuscitation, a newly developed resuscitation method for human patients who do not respond to conventional cardiopulmonary resuscitation^5^,^27^,^28^,^29^, provides relatively consistent blood flow to individual organs compared to conventional CPR, therefore reliably resuscitates animals following prolonged CA^24^.

Rats were randomized into two groups and received either a control diet or DHA-supplemented diet for 7 to 8 weeks. High performance liquid chromatography-mass spectrometry (HPLC-MS) analysis showed significantly altered phospholipid composition following dietary treatment. Subsequently, rats were subjected to 20 min asphyxial CA and 30 min CPB resuscitation. The survival of rats was surveyed through hemodynamic monitoring for up to 4 h after the start of CPB resuscitation. The results show that dietary DHA significantly enhances rat survival in this CA model.

## Results

### Base Line Rat Physiology

We first examined baseline rat physiology (Table 1). DHA treatment did not affect body weight (456 g ± 27 CON vs. 452 g ± 29 DHA). Furthermore, DHA-supplemented diet had no significant effect on hemodynamics as pulse pressure (PP), mean arterial pressure (MAP), and systolic pressure (SP) were similar between the two groups. However, animals in the DHA-treated group showed a trend towards slightly lower heart rate (HR) compared to control animals. Blood analyses indicated acid/base status, gas exchange, and glucose/lactate levels in blood were similar between the two groups (Table 1). Overall, the data indicate that the dietary conditions used for this study had minimal effect on baseline rat physiology.

### Phospholipid Alteration by DHA Supplement

**Figure 1:**
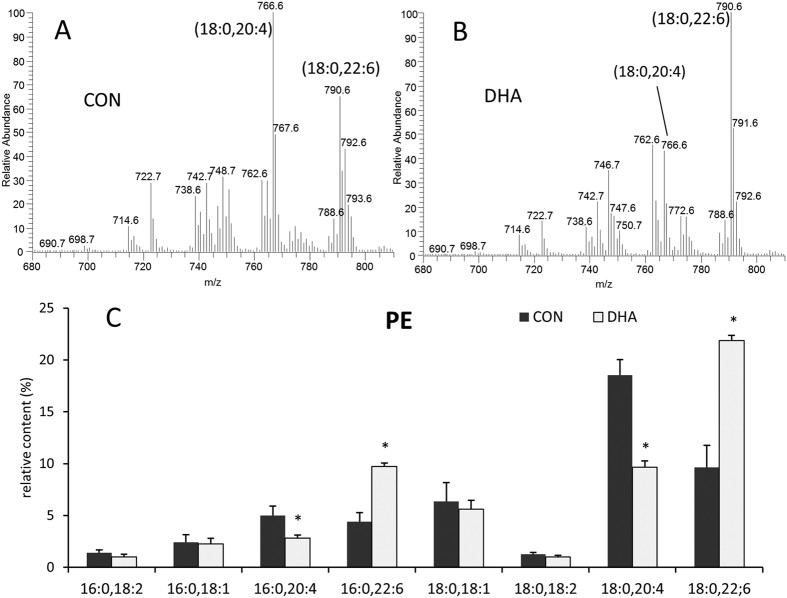
Representative mass spectra of heart phosphatidylethanolamine (PE) extracted from control group (**A**) and DHA-treated group (**B**) as well as the relative content of eight major PE species in both groups (**C**). DHA, 22:6; arachidonic acid (ARA), 20:4; linoleic acid, 18:2; oleic acid, 18:1; stearic acid, 18:0; palmitic acid, 16:0; n = 5.

A DHA-enhanced diet has been shown to increase the phospholipid level of DHA in various tissues^30^,^31^,^32^. In particular, phosphatidylethanolamine (PE) from cardiac tissue is one of the most significantly modified phospholipids^33^. The mass spectra of PE in the control (Fig. 1A) and DHA-treated (Fig. 1B) groups as well as the content of major PE species (Fig. 1C) were analyzed. Animals in the DHA-treated group showed substantial increases in PE species containing DHA, such as PE(16:0,22:6) and PE(18:0,22:6), while species containing ARA, PE(16:0,20:4) and PE(18:0,20:4) were decreased (Fig. 1C). There was no difference in the levels of species containing other major fatty acids, such as linoleic acid (18:2) or oleic acid (18:1). These data show that dietary DHA enhances the PE levels of DHA and this increase is predominantly at the expense of ARA, consistent with previous reports that DHA and ARA compete with one another for incorporation into membrane phospholipids^33^,^34^.

### Survival of Rats

**Figure 2:**
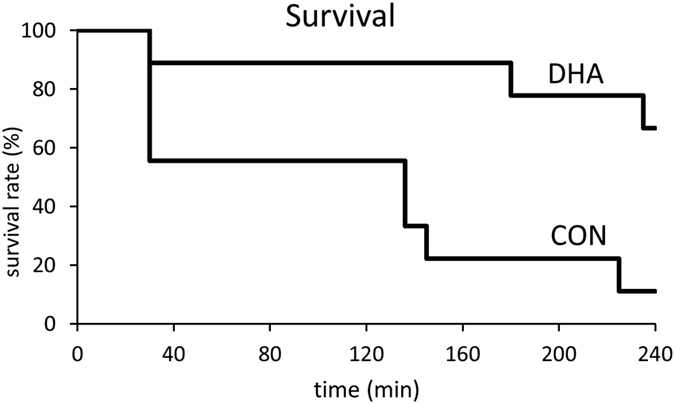
Survival of rats following 20 min CA and 30 min CPB resuscitation. Time of death for rats who did not achieve ROSC was recorded at 30 min following the start of CPB resuscitation (*p* < 0.0001, n = 9).

Following 20 min CA, rat survival was monitored for a total of 4 h following the initiation of CPB resuscitation (Fig. 2). The time of death for rats who did not achieve return of spontaneous circulation (ROSC) was recorded as 30 min. In the control group, 5 of 9 rats achieved ROSC and of the surviving rats, 4 of 5 rats died between 2–3.5 h after CPB resuscitation. In the DHA-treated group, 8 of 9 rats achieved ROSC. All rats who achieved ROSC in the DHA-treated group survived for 3 h and 2 rats died between 3–4 h. Altogether, only 1 of 9 rats survived for 4 h in the control group whereas 6 of 9 rats survived in the DHA-treated group (*p* < 0.0001). This result shows that dietary DHA supplementation significantly increased the short term survival of rats following 20 min CA.

### Hemodynamics

The cardiorespiratory parameters of rats, which achieved ROSC (5 rats in the control group and 8 rats in the DHA-treated group) was compared. As all rats survived for 120 min (Fig. 2) and DHA rats developed seizures after 120 min (see below), we compared HR, MAP, PP, and SP between the two groups until 120 min after the start of CPB resuscitation. The time to reach CA (192 ± 13 sec CON vs. 187 ± 25 sec DHA) and time to achieve ROSC (288 ± 103 sec CON vs. 334 ± 172 sec DHA) were similar between the two groups. Therefore, the rats in both groups used for this comparison experienced a similar duration of ischemia.

**Figure 3:**
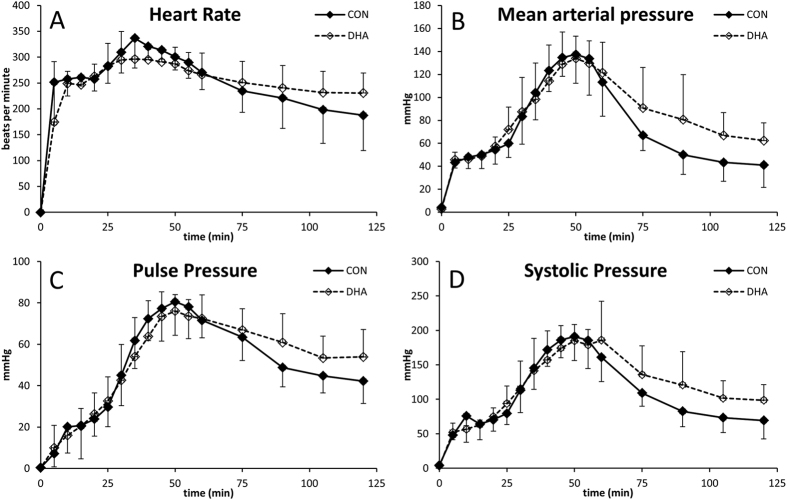
Changes in heart rate (**A**), mean arterial pressure (**B**), pulse pressure (**C**), and systolic pressure (**D**) following the initiation of CPB resuscitation in control and DHA-treated groups (n = 5 for control group and n = 8 for DHA-treated group).

As shown in Fig. 3, all parameters of the rats in the both groups gradually increased until 50 min, after which a decrease was observed. After 50 min, cardiorespiratory parameters of rats in the control group decreased more rapidly than those in the DHA group. Following 120 min, HR, MAP, PP, and SP of rats in the DHA-treated group were higher than rats in the control group by 20–30%, suggesting that dietary DHA improved heart function at this early stage of recovery following 20 min severe CA and 30 min CPB resuscitation. These enhanced cardiorespiratory parameters should be important for survival as well as the recovery of other organs.

### Brain Function

Following 20 min CA and 30 min CPB resuscitation, all rats in the control group were unconscious and lacked spontaneous movement. Rats also showed no response to toe pinch or corneal stimulation. From our previous experiments studies varying CA times, toe pinch and corneal reflex were the only stimuli animals responded to following severe CA. Therefore, a lack of response to these stimuli was evidence of complete loss of motor responses. Overall, 20 min CA placed rats in the control group into a deep unresponsive coma, a criterion for brain death^35^.

**Figure 4:**
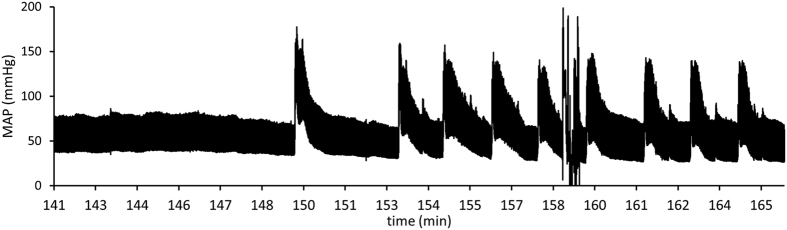
Representative changes in mean arterial pressures (MAP) showing recurrent seizures in a DHA-treated rat following 20 min CA and 30 min CPB resuscitation.

Rats in the DHA-treated group also did not respond to toe pinch or cornel stimulation. However, surviving rats in the DHA group started developing recurrent seizures 2 h following the initiation of CPB resuscitation. These seizures caused visible whole-body movements and were also detectable by abrupt increases in MAP (Fig. 4). After 180 min, all surviving rats in the DHA-treated group displayed recurrent seizures, which continued until death or 4 h of survival was reached. Rats also resumed spontaneous respiration, evidenced by ventilator dyssynchrony. Recurrent seizures and spontaneous respiration shows that surviving rats in the DHA group preserved a certain level of brain function and were not brain dead^35^. However, in the control group, none of the five surviving rats developed seizures until 135 min. Only one rat, which survived for 4 h, displayed recurrent seizures after 220 min. These observations suggest that dietary DHA protected the brain during CA and resuscitation.

### Blood Gas Analysis

The arterial blood gas was analyzed at 60 and 135 min after the initiation of CPB resuscitation (Table 2). Compared to baseline measurements, animals in both groups had decreased pH levels (7.24 from 7.42 CON; 7.28 from 7.43 DHA) and increased lactate levels (4.2 from 1.3 mmol/L CON; 3.5 from 1.2 mmol/L DHA) after 60 min. This was most likely a consequence of increased anaerobic metabolism, a common phenomenon under ischemic conditions^36^. The acidosis became more severe after 135 min, where the average pH further decreased to 7.14 in the control group and 7.11 in the DHA-treated group, and lactate levels increased to 6.8 mmol/L and 7.3 mmol/L, respectively (Table 2). At the end of the 4 h survival, the average glucose level increased to 192 mg/dL, the lactate level decreased to 3.91 mmol/dL, and pH increased to 7.25 in the surviving animals in the DHA-treated group.

All blood gas parameters were similar at 60 min after the initiation of CPB resuscitation between the two groups. There was a trend towards slightly decreased glucose and lactate levels in the DHA-treated group, although this lacked statistical significance. At 135 min, the lactate levels remained similar between the two groups (6.8 mmol/L CON vs. 7.3 mmol/L DHA), however, the trend in lower glucose levels in DHA-treated animals became more pronounced (204 mg/dL CON vs.133 mg/dL DHA, *p* = 0.12). The pO_2_ level was significantly higher by 50% (131 mmHg CON vs. 261 mmHg DHA, *p* = 0.02) in DHA-treated rats compared to animals in the control group. The lower glucose levels suggested that animals in the DHA-treated group had increased glucose metabolism. In general, blood gas parameters were in agreement with metabolic disorders associated with ischemia.

## Discussion

We have demonstrated that rats on a DHA-enriched diet have a significantly higher survival rate following 20 min CA compared to rats on a standard diet. Animals in the DHA-treated group exhibited a higher success rate in achieving ROSC and improved hemodynamic performance at the early stage of recovery following CPB resuscitation. In addition, recurrent seizures and spontaneous breathing were observed in all rats in the DHA group compared to the lack of any brain function in the majority of control animals, suggesting that dietary DHA helps in preserving brain function. Taken together, the data suggest that improved heart and brain function by dietary DHA supplementation may contribute to enhanced short-term survival found in this study. However, it may be possible that seizures, the sign of brain function remaining, may eventually be detrimental to long term survival.

Blood gas levels in the DHA-treated rats at 130 min were also consistent with the observed improved survival and physiological outcomes. The decreased blood glucose levels in the DHA-treated group suggest that glycolysis is more efficient in these animals relative to the control group. Despite the presumed increased consumption of glucose in the DHA-treated group, the lactate levels were similar to the control group, suggesting an improved restoration of oxidative pyruvate metabolism in the DHA-treated animals. As glycolytic aerobic metabolism is a central pathway for energy production in the brain and heart, this result is consistent with enhanced heart and brain function.

A more dramatic difference is found in the blood oxygen concentration, which was significantly higher (50%, *p* = 0.02) in the DHA-treated animals compared to the control group. Based on the observation that 3 of the 5 animals in the control group died within 20 min following blood collection, lower oxygen concentration in control animals may have resulted from impared gas exchange due to lung injury. However, this hypothesis was not supported due to the lack of difference in the partial pressure of carbon dioxide (pCO_2_) and the concentration of bicarbonate (HCO_3_^−^) between the two groups. Rather, it suggests that increased oxygen utilization may be the reason for the lower blood oxygen concentration observed in control animals.

As discussed above, oxygen utilization in the control group is not likely due to aerobic metabolism. Instead, phospholipid analysis provides a possible explantion for this increased oxygen consumption in control animals. Ischemia activates phospholipase A2 and many isoforms of phospholipase A2 preferentially hydrolyze ARA^37^,^38^,^39^,^40^,^41^. The resulting free ARA is then further metabolized by oxidative enzymes, such as lipoxygenase and cyclooxygenases, in an oxygen-dependent mechanism^10^. The increased products from the oxidation of ARA are commonly found in various ischemic models^42^,^43^. Therefore, replacing ARA with DHA in phospholipids should reduce the metabolism of ARA and thus reduce oxygen consumption.

This reduced ARA metabolism also provide two potential protective mechanisms for dietary DHA. Firstly, as many isoforms of phospholipase A2 preferentially hydrolyze ARA, the replacement of ARA with DHA in phospholipids should result in phospholipids becoming more resistant to hydrolysis, preventing the decomposition of membrane phospholipids during ischemia/reperfusion. Secondly, metabolites of ARA are mostly pro-inflammatory^44^. Therefore, replacing ARA in the lipid pool could reduce the generation of inflammatory metabolites and thus reduce inflammatory reactions post-resuscitation. In fact, acute admistration of DHA has been shown to reduce the levels of such ARA metabolites^45^.

In this context, studying the effect of dietary DHA requires consideration of the amount of ARA consumed by subjects as well. This study and literature support the notion that DHA and ARA compete with one another for the limited lipid pool^10^,^33^. Since ARA is rich in common diets, such as those consisting of chicken and eggs, the net uptake of DHA in the lipid pool could be affected by the consumption of ARA-rich diets. Therefore, the amount of DHA intake may not account for the protective effect of dietary DHA, but rather the relative amount of DHA compared to ARA consumed in our daily meal. This may explain the inconsistent results found in human studies on the effects of dietary DHA^11^,^12^,^13^,^14^,^15^,^16^,^17^ and emphasizes the importance of the Omega-3 index as a necessary control in assessing the effects of dietary DHA^46^.

A limitation in this study is the absence of long-term survival and direct assement of heart and brain function. As 20 min CA causes highly severe injury, animals die within a few minutes of extubation. We chose this severe injury model in an attempt to maximize the protective effects of DHA. To further explore the protective effects of DHA on the brain, DHA should be tested in less severe injury models, where long-term survival can be achieved. How the metabolism of DHA and ARA is affected by the DHA-supplementation should also be further assessed. Correlation between changes in the metabolism of these fatty acids and survival will provide insights into the role that fatty acids play during ischemia and resuscitation.

In summary, the data show that dietary DHA significantly improves survival in rats following 20 min CA and 30 min CPB resuscitation. Improved hemodynamics observed in DHA-treated rats indicat that dietary DHA protects the heart at this early stage of recovery.

## Methods

### Animals and diets

The animal protocol was approved by the Institutional Animal Care and Use Committee of the Children’s Hospital of Philadelphia. All experiments were performed according to the approved protocol. Male Sprague–Dawley rats (150–172 g, Charles River) were randomized into two groups, receiving either a control diet (2016 Teklad Global 16% Protein Rodent Diet, Harlan Laboratory) or a DHA-supplemented diet, formulated by mixing the control diet with DHA-rich oil to give 1.8% of total energy from DHA (Harlan Laboratory). The DHA-rich oil (~70%) was a generous gift from Croda. Diet treatment was maintained for 7–8 weeks with unrestricted access to food and water.

### Asphyxial CA and CPB Resuscitation

Detailed procedures for asphyxia and CPB resuscitation are described in supplementary information^25^. Briefly, rats were anesthetized with 4% isoflurane and placed on a thermostatically regulated heating pad. Rat temperature was kept at ~37 °C using the heating pad and a heating lamp throughout the procedures. Rats were mechanically ventilated and anesthesia was maintained with 1–2% isoflurane. Buprenorphine was injected to reduce pain in animals. After surgical preparation, vecuronium bromide at 2 mg/kg was administered through the left femoral vein. CA was induced by stopping the ventilator and discontinuing isoflurane. After 20 min asphyxia, resuscitation was started with the initiation of CPB flow and resumption of ventilation. Animals were initially ventilated with 100% oxygen and 60% oxygen after 90 min. CPB resuscitation was continued for 30 min and survival was monitored for an additional 3.5 h.

### Hemodynamics, Blood Gas Analysis, and Survival Outcomes

Hemodynamic monitoring was used to measure MAP, HR, PP, and SP. The time to reach CA and the time of death was determined based on an MAP below 20 mmHg^25^. Death was defined by MAP below 30 mmHg lasting for 5 min. For blood gas analysis, 0.15 mL blood samples were drawn from the left femoral catheter at baseline and designated times following CPB resuscitation.

### Phospholipid Analysis

To assess the dietary effects of DHA on the phospholipid composition, PE was extracted from the heart and analyzed following a previously published method^47^,^48^. Briefly, heart tissue was pulverized under liquid nitrogen and extracted for lipids using Christianson’s method^49^. Phospholipids were separated from the lipid mixture by solid phase extraction. HPLC-MS data were obtained with a LTQ Velos spectrometer (Thermo Scientific) and processed using X-calibur software (Version 2.2) following previously reported methods^47^,^48^.

### Statistical Analysis

Rat survival between the two groups was compared using the Wilcoxon method. Data from phospholipid, cardiac function, and blood gas analyses were presented as mean ± standard deviation, and significance was tested using Student’s t-test. A two-tailed *p* value < 0.05 was considered statistically significant.

## Additional Information

**How to cite this article**: Kim, J. *et al*. DHA-supplemented diet increases the survival of rats following asphyxia-induced cardiac arrest and cardiopulmonary bypass resuscitation. *Sci. Rep.* **6**, 36545; doi: 10.1038/srep36545 (2016).

**Publisher’s note:** Springer Nature remains neutral with regard to jurisdictional claims in published maps and institutional affiliations.

## Supplementary Material

Supplementary Information

## Figures and Tables

**Table 1:** Body mass, baseline hemodynamics, and blood gas (n = 9).

	CON	DHA
Body weight (g)	456 ± 27	452 ± 29
Body temperature (°C)	36.7 ± 0.4	36.8 ± 0.6
Cardiac function		
Heart rate (bpm)	330 ± 38	303 ± 43
Mean arterial pressure (mmHg)	88.7 ± 11.4	88.6 ± 15.7
Systolic pressure (mmHg)	123 ± 20	125 ± 21
Pulse pressure (mmHg)	52.7 ± 15.0	54.9 ± 11.9
Blood gas		
pH	7.42 ± 0.05	7.43 ± 0.03
pO_2_ (mmHg)	101.5 ± 27.3	99 ± 26.7
pCO_2_ (mmHg)	33.3 ± 5.4	31.7 ± 3.9
HCO_3_ (mEq/L)	20.9 ± 2.0	21.1 ± 1.9
TCO_2_ (mmol/L)	23.0 ± 2.0	22.0 ± 1.9
SaO_2_ (%)	97.3 ±1.9	97.4 ± 2.1
Hematocrit (%)	37.8 ±2.0	37.0 ± 2.7
Glucose (mg/dL)	217 ± 46	232 ± 57
Lactate (mmol/L)	1.3 ± 0.3	1.2 ± 0.4

**Table 2:** Blood gas analysis following CA and CPB resuscitation (n = 5 for CON and n = 8 for DHA).

	60 min		135 min		240 min
	CON	DHA	CON	DHA	DHA^1^
Blood gas					
pH	7.24 ± 0.04	7.28 ± 0.03	7.14 ± 0.13	7.11 ± 0.10	7.25 ± 0.02
pO_2_ (mmHg)	293 ± 52	328 ± 57	131 ± 56*	261 ± 82	162 ± 102
pCO_2_ (mmHg)	44.9 ± 4.5	40.5 ± 4.2	44.8 ± 8.1	48.9 ± 5.6	39.6 ± 8.1
HCO_3_ (mEq/L)	19.1 ± 2.0	19.0 ± 2.0	15.5 ± 4.7	15.7 ± 3.6	17.2 ± 2.9
TCO_2_ (mmol/L)	20.4 ± 1.8	20.1 ± 2.0	17.6 ± 4.3	17.2 ± 3.7	18.5 ± 3.1
SaO_2_ (%)	100	100	92.4 ± 12.6	99.6 ± 0.9	98.7 ± 2.1
Hematocrit (%)	34.5 ± 1.9	33.7 ± 3.7	36.5 ± 3.4	36.5 ± 3.0	35.6 ± 3.5
Glucose (mg/dL)	143 ± 33	118 ± 37	204 ± 66^†^	133 ± 31	192 ± 85
Lactate (mmol/L)	4.2 ± 1.2	3.5 ± 0.7	6.8 ± 3.5	7.3 ± 4.1	3.9 ± 1.1

^1^Average of 6 surviving rats in the DHA-treated group; *p = 0.02; ^†^*p* = 0.12.
