# Supplementary material for: DHA-supplemented diet increases the survival of rats following asphyxia-induced cardiac arrest and cardiopulmonary bypass resuscitation
Source: Sci Rep. 2016 Nov 4;6:36545. doi: 10.1038/srep36545 (PMC5109906; doi:10.1038/srep36545)
Supplement: Supplementary Information [file srep36545-s1.pdf]

Supporting Information to

DHA-supplemented diet increases the survival of rats following asphyxia-induced cardiac arrest and cardiopulmonary bypass resuscitation

Junhwan Kim*, Tai Yin, Koichiro Shinozaki, Joshua W. Lampe, and Lance B. Becker

Department of Emergency Medicine, Feinstein Institute for Medical Research, Northwell Health System, Manhasset, New York, 11030, United States of America.

*corresponding: jkim46@nshs.edu

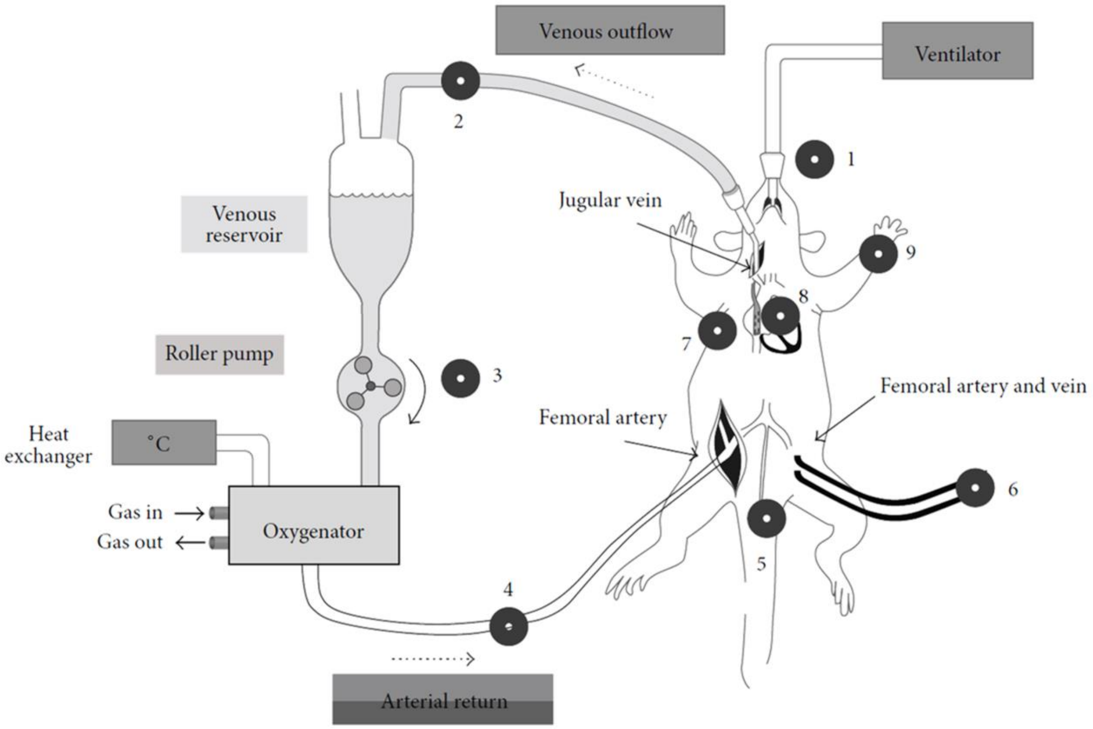

1. Capnograph; 2. Oxygen saturation and hematocrit (Critline); 3. Pump; 4. Temperature, PO_2 and system pressure of arterial return; 5. Rectal temperature; 6. Arterial & central venous blood pressure; 7. Electrocardiography; 8. Esophageal temperature; 9. Pulseoximetry

Figure S1. Diagram showing surgical procedure of asphyxial CA and CPB resuscitation.

Surgical procedure for asphyxia and CPB resuscitation. Rats were anesthetized with 4% isoflurane for 5min in a plexiglass chamber and orotracheally intubated with a 14-gauge intravenous catheter (B. Braun Medical Inc., Bethlehem, PA, USA). Rats are placed on a thermostatically regulated heating pad and rat temperature was kept at 37 °C using the heating pad and a heating lamp during the procedures. Ventilation was adjusted to maintain an EtCO2 between 35 and 45mmHg (Micro-Capnometer, Columbus Instruments, Columbus, OH, USA), a respiratory rate (RR) of 40 per min, and a positive end-expiratory pressure (PEEP) of 1 cm H2O using a mechanical ventilator (Ventilator Model 683, Harvard Apparatus, Holliston, MA, USA). Anesthesia was maintained with 2% isoflurane to sustain a surgical depth of anesthesia.

Temperatures were monitored using tympanic and rectal thermocouple probes and were maintained steadily at 37±0.5 ◦C with a heat pad and an incandescent heating lamp.

After abdominal injection of buprenorphine (0.05 mg/kg), left femoral artery and vein were cannulated with polyethylene catheters to measure arterial and central venous pressure, respectively. The right external jugular vein and the right femoral artery were cannulated for venous outflow and the arterial inflow of CPB fluid. After surgical preparation, which takes an average of 1h, isoflurane was decreased to 1% and vecuronium bromide at 2 mg/kg was slowly administered through the left femoral vein . Asphyxial cardiac arrest was induced by switching off the ventilator. Isoflurane was discontinued thereafter.

After 20 min of asphyxia, resuscitation was started with the initiation of CPB flow and resumption of ventilation. The customized CPB circuit designed for rodents consists of a heat exchanger, an open venous reservoir, a membrane oxygenator, and a roller pump. The open venous reservoir was filled with a ~20 mL solution cocktail of plasmalyte A (10 mL), 6% hetastarch (10 mL), 5% magnesium sulfate (0.8 mL), and 3.3 M THAM (0.3 mL) and 10 mL of blood from a donor rat. For the oxygenator, 100% oxygen was used to saturate exiled blood with oxygen. The initial CPB flow rate was 70 mL/min and gradually decreased to ∼20 mL/min to meet venous outflow. After 30 min, CPB flow was stopped. Ventilation was subsequently adjusted to a $PaCO_2$ of 35 to 45 mm Hg. Initially, animals were ventilated with 100% oxygen. The oxygen concentration was decreased to 60% after 90 min.
